# Multiple Mechanisms Increase Levels of Resistance in *Rapistrum rugosum* to ALS Herbicides

**DOI:** 10.3389/fpls.2016.00169

**Published:** 2016-02-22

**Authors:** Zahra M. Hatami, Javid Gherekhloo, Antonia M. Rojano-Delgado, Maria D. Osuna, Ricardo Alcántara, Pablo Fernández, Hamid R. Sadeghipour, Rafael De Prado

**Affiliations:** ^1^Department of Agronomy, Gorgan University of Agricultural Sciences and Natural ResourcesGorgan, Iran; ^2^Department of Agricultural Chemistry and Soil Science, University of CórdobaCórdoba, Spain; ^3^Agrarian Research Center “Finca La Orden” ValdesequeraBadajoz, Spain; ^4^Department of Biology, Golestan UniversityGolestan, Iran

**Keywords:** *Rapistrum rugosum*, ALS-inhibiting herbicides, dose-response, ^14^C-TM, TSR and NTSR-mechanisms

## Abstract

*Rapistrum rugosum* (turnip weed) is a common weed of wheat fields in Iran, which is most often controlled by tribenuron-methyl (TM), a sulfonylurea (SU) belonging to the acetolactate synthase (ALS) inhibiting herbicides group. Several cases of unexplained control failure of *R. rugosum* by TM have been seen, especially in Golestan province-Iran. Hence, there is lack of research in evaluation of the level of resistance of the *R. rugosum* populations to TM, using whole plant dose-response and enzyme assays, then investigating some potential resistance mechanisms Results revealed that the resistance factor (RF) for resistant (R) populations was 2.5–6.6 fold higher than susceptible (S) plant. Neither foliar retention, nor ^14^C-TM absorption and translocation were the mechanisms responsible for resistance in turnip weed. Metabolism of TM was the second resistant mechanism in two populations (Ag-R5 and G-1), in which three metabolites were found. The concentration of TM for 50% inhibition of ALS enzyme activity *in vitro* showed a high level of resistance to the herbicide (RFs were from 28 to 38) and cross-resistance to sulfonyl-aminocarbonyl-triazolinone (SCT), pyrimidinyl-thiobenzoate (PTB) and triazolopyrimidine (TP), with no cross-resistance to imidazolinone (IMI). Substitution Pro 197 to Ser 197 provided resistance to four of five ALS-inhibiting herbicides including SU, TP, PTB, and SCT with no resistance to IMI. These results documented the first case of *R. rugosum* resistant population worldwide and demonstrated that both RST and NRST mechanisms are involved to the resistance level to TM.

## Highlight

*Rapistrum rugosum* (L.) is a common weed of wheat and rapeseed in Iran, which is most often controlled by tribenuron-methyl (TM).

For years, this herbicide has been used for controlling this weed but recently there have been some reports indicating unexplained control failure of *R. rugosum* by TM, especially in the wheat fields.

Different TM resistance levels between the *R. rugosum* populations were found. ALS mutation (Pro197Ser) is likely to be the cause of the cross resistance found in those populations to four of the five families of the ALS inhibitors group. Moreover, highest levels of resistance, at whole plant level, were found in those populations that showed two different mechanisms of resistance: enhanced metabolism and punctual ALS mutations.

These results documented the first case of *R. rugosum* resistant population worldwide and demonstrated that the addition of resistant site target and non-resistant site target mechanisms are responsible to the highest resistance level to TM.

## Introduction

Extended use of herbicideapplications will inevitably result in a rapid evolution of weeds with herbicide resistance. This has meant that weed researchers challenges and those interested in inventory control techniques have faced many challenges. Actually, while talking about the chemical control of weeds, three main issues should be highlighted: first is the weeds’ resistance to the action mechanisms of available herbicide; second, the negative impact of the regulatory and economic means in phasing out the older herbicides, and in particular some specific herbicide mechanisms of action (MOA); and third, the inaccessibility of new herbicides, especially herbicides with a new mode of action (Ruegg et al., 2007). The application of most of the known herbicides causes enzyme inhibition, and just a few of them disrupt other processes such auxin response or cell division. This limits the herbicide targets to a few groups of the plant genes. It has resulted in the increase in number of resistant weed species, which along with the absence of new herbicides, have made the traditional chemical weed control programs largely ineffective. For instance, there are now 461 cases of herbicide-resistant biotypes in 247 species covering all herbicide modes - of – action (Heap, 2015). It would seem that, in the near future, there will be inadequate chemical control methods for several weed species of the major row crops (Stewart, 2009).

Generally speaking, mechanisms of resistance to herbicides can be divided into two groups: target-site (TSR) and non-target-site mechanisms (NTSRs). Target-site resistance occurs due to mutations in the genes of the encoding proteins, when they are targeted by the herbicide (e.g., changing the binding sites of herbicide) or by overproduction of the target enzyme (gene overexpression or amplification). On the other hand, in non-target site resistance, there are no significant changes at the protein sequence or protein expression level, although this subject is more complicated and less known in both a biological and genetic context. In any case, it might be said that, theoretically, non - target - site resistance could minimize the amount of active herbicide reaching the target site (e.g., decreased foliar uptake or translocation out of treated sections, increased herbicide sequestration, or enhanced herbicide metabolism; Yuan et al., 2007). Enhanced herbicide metabolism in weeds is usually found by measuring ^14^C-herbicide and its ^14^C-metabolites in intact plants in the absence and presence of cytochrome P_450_ inhibitors (De Prado et al., 2005; Yasuor et al., 2010). The major metabolites are identified by TLC or HPLC using standard non-toxic metabolite-derived herbicide itself (Christopher et al., 1992; De Prado et al., 2005). A few cases of resistance-metabolism to acetolactate synthase (ALS)-inhibitor herbicides have been detected and studied in weed species (Yu and Powles, 2014a,b).

It is obvious that the most widely made use of the herbicide will result in the rapid evolution of resistant weeds. An early detection of herbicide resistance in a weed biotype suspected of resistance requires a series of tests that could show plant response to different doses of herbicide. These experiments should study the response of whole plants suspected of resistance rather than susceptible biotypes in the range of herbicide doses in a greenhouse assay. A dose-response experiment is often conducted to determine the level of resistance and obtain a glimpse of a potential resistance mechanism.

As there are positive attitudes and efforts toward control of the spread of resistance in the plants, it seems quite necessary to gain better and more comprehensive understanding of the resistance mechanisms. Discovering the basic mechanisms of resistance is the first step in the attempt to solve problems and develop novel remedies for resistant weed management in agricultures (Stewart, 2009). However, many more efforts must be made in this direction.

Thus, it has been noted that there are many cases of ALS inhibitor-resistance in rice and wheat because many of the ALS inhibitor resistant weeds in corn, soybean, and cotton can be controlled by glyphosate in Roundup Ready crops. Actually, it has been proved that ALS is the common target site of five herbicide chemical groups: sulfonylurea (SU), imidazolinone (IMI), triazolopyrimidine (TP), pyrimidinyl-thiobenzoates (PTB), and sulfonyl-aminocarbonyl-triazolinone (SCT).

There are evidence that prove the resistance to ALS-inhibiting herbicides allege that this most often results from a single amino acid substitution in the ALS enzyme as a target based in mechanism of resistance. There are five highly conserved amino acids in the ALS gene. In every case investigated, target-site resistance to ALS-inhibiting herbicides has been attributed to a change in one of the eight amino acids located in these regions including Ala-122, Pro-197, Ala-205, Asp-376, Arg-377, Trp-574, Ser-653, and Gly-654. Substitutions in these ALS amino acids have been implicated in herbicide resistance in field selections from natural weed populations with target-site resistance. Mutations in these eight amino acids disrupt herbicide binding thus converting the susceptible enzyme into the one resistant to the herbicide (Corbett and Tardif, 2006). Different mutations result in various cross-resistance patterns. For example, the Trp- 574-Leu substitution confers resistance to all families of ALS-inhibiting herbicides in many weed species, while the Ala-122-Thr substitution endows weed resistance to IMI but not to SU herbicides (Powles and Yu, 2010; Beckie et al., 2012). Few cases have been found in which the accumulation of TSR and NTSR increases resistance mechanisms in plants. The only known and well-studied case has been a population of *Lolium rigidum* found resistant to chlorsulfuron after seven consecutive years of treatment with low doses of herbicide in Australia. Only 4% of individuals of this population had two mechanisms of resistance, enhanced rates of chlorsulfuron metabolism and ALS mutations the rest are only possesses metabolism and they are individuals with weak resistance level to chlorsulfuron (Christopher et al., 1992; Burnet et al., 1994; Yu et al., 2008).

Recently, *Rapistrum rugosum* (L.) resistance has been reported by Derakhshan and Gherekhloo (2012). It is a common weed of wheat and rapeseed in Iran, which is most often controlled by TM. For years, this herbicide has been used for controlling this weed but recently there have been some reports indicating unexplained control failure of *R. rugosum* by TM, especially in the wheat fields. First case of *R. rugosum* resistant to ALS-inhibitors (chlorsulfuron) was detected in Australia in 1996, but mechanisms responsible of this resistance were not studied (Adkins et al., 1997). The main objective of this work was a survey of *R. rugosum* resistance in Iran wheat fields, and the specific objectives to determine: (1) the level of TM resistance of the different populations detected (2) the resistance mechanisms involved and (3) the molecular pattern of ALS resistance determined and thereby distinguish the resistant populations and cross resistance motif.

## Materials and Methods

### Seed Source

Suspected resistant seeds used in this study were collected from the Golestan province of Iran in the spring of 2011, 2012, 2013, and 2014. The suspected seeds were collected and bulked from the fields with the following characteristics: first, fields with a history of repeated leaf-applied TM herbicide use for 4 or 5 successive years. Second, the field where the farmers were not satisfied with the efficiency of the herbicide in their wheat fields so that after using it, the field was with this weed. The susceptible reference populations were collected from the no-herbicides infected fields. All seeds were air-dried and stored in paper bags at 4°C until use in experiments.

### Screening Test

For primary screening of a suspicious resistant population, the experiment was arranged in a completely randomized design with three replications (one pot per replicate). After breaking the seed dormancy [seeds were floated in 2000 ppm gibberellic acid (GA) for 24 h then kept in Petri dishes containing moist filter paper for 24 h in a refrigerator at 4°C in the dark], five germinated seeds were planted in suitable plastic pots. Four weeks after planting, at the three- to four-leaf stage, TM was applied at the recommended rate of 15 g a.i. ha^-1^ using a calibrated sprayer with a flat-fan nozzle (TeeJet 8001) to deliver 250 L ha^-1^ of spray solution at 200 kPa. One untreated control for each seed population was considered. Plants were harvested 4 weeks after herbicide application and the dry shoot weight was recorded. To examine the differences between populations, data were expressed as percentages of untreated control. Also, the numbers of dead and surviving plants were counted and visual phytotoxicity rating was scored according to the EWRC method (European Weed Research Council; 1 = completely inhibition, 9 = no effect; Sandral et al., 1997). Data were subjected to analysis of variance (ANOVA), and comparison of means based on a LSD test procedure at the 0.05 significance level. Due to presence of zero values in the original data, we have performed logarithmic data transformation prior to ANOVA. Then, the original data were displayed for means comparison in the table.

### TM Plant Response

To consider the resistance factor (RF), the seed sources presenting 50% of surviving plants or with 80% relative dry weight of the untreated control in the screening test were selected.

The dormancy seeds have been broken and then were sown in suitable pots and placed in the greenhouse under natural sunlight (winter 2014 at Gorgan university). Plants with three to four true leaves were sprayed using a standard and calibrated sprayer as previously mentioned. TM rates were based on a logarithmic scale of 0, 0.25, 0.5, 1, 2, 4, 8, 16, and 32 times the recorded post-emergence commercial rate of utilization.

Above-ground biomass from the plants in each pot was harvested 4 weeks after treatment (WAT) and dried at 60°C for 48 h. Dry biomass data were expressed as a percentage of the untreated control within each population.

### TM Spray Retention

This assay was performed according to methodology described by Gauvrit (2003). A solution of 15.0 g TM ha^-1^ (Granstar 75 WG) mixed with a solution of 100 mg L^-1^ of fluorescein in 10 ml of 5 mM NaOH was applied on plants at the three to four-leaf stage in a laboratory spray chamber equipped with a flat fan nozzle (TeeJet 8002 EVS) calibrated to deliver 250 L ha^-1^ at a pressure of 200 kPa.

After the impregnated solution had dried on the foliage (>10 min), the plants were cut off at ground level, and immersed for 30 s in 50 ml 5 mM NaOH to wash the tissue. The tissues were dried in an oven at 60°C for 72 h. The absorbance of fluorescein was measured using a spectrofluorometer at 490 exc/510 emi nm. The retention was expressed in μL of TM solution g^-1^ dry matter. The experiment was conducted in a completely randomized design using seven plants from each population as replications. Data obtained were subjected to ANOVA, and comparison of means based on an LSD procedure at the 0.05 significance level with Statistix 9.0.

### ^14^C-TM Absorption and Translocation

Resistant and susceptible plants of four *R. rugosum* populations were grown as described above. An herbicide solution was prepared with commercial products based on the recommended dose (15 g ha^-1^ dissolved into 250 L ha^-1^) and mixed with ^14^C-TM to prepare an emulsion with a specific activity of 833.33 kBq μL^-1^. This emulsion was applied to the surface of the second expanded leaf from each population in 1 μL droplet using a microapplicator (Hamilton PB 6000 dispenser, Hamilton, Co., Reno, NV, USA). At 24, 48, 72, and 96 h after application (HAT) plants were harvested in batches of three plants and separated into treated leaf (TL), remainder of shoots (RS), and roots (Ro). The surface of the treated leaves was washed with 1 mL of acetone plus water (1:1 V/V) solution. The washes from each batch were mixed with 2 mL of scintillation fluid (Ultima Gold^TM^; Perkin-Elmer, Packard Bioscience BV), and the radioactivity was determined by liquid scintillation spectrometry (LSS; Beckman LS 6000 TA, Beckman Instruments, USA). The plant sections were stored individually in combustion cones (Combuste-Cone, Flexible: Perkin-Elmer, Packard Bioscience BV), dried at 60°C for 48 h and combusted in a sample oxidizer (Packard 307, Packard Instruments, Meriden, CT, USA). The released ^14^CO_2_ was trapped and counted in 18 mL of Permafluor and Carbo-Sorb E (1:1, V/V; Perkin-Elmer, BV Bioscience Packard) in scintillation vials. The total radioactivity in the samples was determined by the LSS. The absorbed radioactivity of the herbicide was calculated according to the following formula:

% absorption=[C⁢ in⁢ combusted⁢ tissue14(C⁢ in⁢ combusted⁢ tissue+C⁢ in⁢ leaf⁢ washes1414)]×100.

The translocation of ^14^C-TM was expressed as a percent from absorbed radioactivity. It was expressed in each part of the plant, with respect to the total radioactivity present within the plant after 96 h. The experiment was repeated twice.

The ^14^C-tribenuron-methyl translocation was visualized using a Phosphor Imaging (Cyclone, Perkin Elmer, Packard Bioscience BV). After the mentioned period of time had passed, whole plants were washed, fixed on filter paper (25 cm × 12.5 cm), dried at room temperature for 4 days, and placed on a film with phosphor crystals (AGFA CURIX) for 6 h. Three plants were used for each population.

### ^14^C-TM Metabolism

Metabolism studies with ^14^C-TM were performed in order to verify whether there were any differences between the different populations of *R. rugosum* that could explain the resistance to tribenuron-methyl detected in this weed (Cruz-Hipolito et al., 2013).

Plants that grew under the same conditions explained before were used and subjected to the same experimental procedure employed for testing absorption and translocation, except that each treated leaf was washed 96 h after application of the labeled herbicide solution to wash away the radioactivity not absorbed on the adaxial surface.

Entire plants were divided into roots and leaf tissues, and stored at -40°C until extraction. Frozen leaf tissues were ground in a mortar with 3 mL of a solution of distilled water + methanol (4:1 v/v). The homogenate residue was washed twice with 3 mL of a solution of methanol + distilled water (4:1 v/v) and placed in a centrifuge at 20000 *g* and 4°C during 10 min (Avanti J-25 with a rotor JA.20, Beckman Instruments, Inc., Fullerton, CA, USA). Both supernatants were mixed and the total radioactivity was quantified with aliquots of 100 μL using a liquid scintillation counter (Beckman LS 6500 TA, Beckman Instruments, Inc., Fullerton, CA, USA).

Mixtures of supernatants were dried at room temperature under a flow rate of liquid nitrogen (0.25 atm). The dry extract was suspended in 200 μL of isopropanol. The ^14^C-tribenuron-methyl and its metabolites was separated by thin layer chromatography (TLC) on a plate 20 cm × 20 cm × 250 cm silica gel (silica gel 60, Merck, Darmstadt, Germany), with isopropanol: ethyl acetate: ammonia: H_2_O (10: 6: 3: 1; v: v: v: v).

The radioactive zones were detected by scanner obtaining radiochromatograms. The radioactivity of the separated products was quantified with a linear analyzer plate equipment (Berthold LB 2821, Wildbald, Germany), while the chemical nature of the separated products was determined by comparison with standards (tribenuron-methyl, metsulfuron-methyl, metsulfuron methyl hydroxylated). The experiment was performed in duplicate, with three plants per replicate sampled and per time.

### ALS Enzyme Activity

Acetolactate synthase activity was measured by determining the formation of the acetoin product after acid decarboxylation of acetolactate in the presence of acid (Osuna and De Prado, 2003). Plant tissue from resistant (R) and susceptible (S) populations was used for the assays and grown under the conditions described previously.

Leaf tissues (3 g) from plants at the 3–5 five leaf stage were frozen with liquid N_2_ and ground using an extraction buffer (1:2 tissue:buffer) containing polyvinylpyrrolidone (PVPP; 0.5 g). The extraction buffer was composed of 1 M K-phosphate buffer solution (pH 7.5), 10 mM sodium pyruvate, 5 mM MgCl_2_, 50 mM thiamine pyrophosphate, 100 μM flavin adenine dinucleotide (FAD), 12 mM dithiothreitol, and glycerol (1:9; v/v). The solution was agitated for 10 min at 4°C. The homogenate was filtered through four layers of cheesecloth and centrifuged (20,000 rpm for 20 min). The supernatant contained a crude ALS enzyme extract, which was immediately used for the enzyme assays.

The ALS activity was assayed by adding 0.09 mL of enzyme extract to 0.11 mL of freshly prepared assay buffer (0.08 M K-phosphate buffer solution [pH 7.5], 0.5 M sodium pyruvate, 0.1 M MgCl_2_, 0.5 mM thiamine pyrophosphate, and 1 μM FAD) containing increasing concentrations of different herbicides. These herbicides were tribenuron-methyl (Sulfonylureas–SU–), bispyribac sodium (Pyrimidinylthiobenzoates–PTB–), flucarbazone (Sulfonylaminocarbonyltriazolinone–SCT–), florasulam (Triazolopyrimidines–TP–), and imazamox (Imidazolinones–IMI–).

A solution of 0.04 M K_2_HPO_4_ (pH 7.0) was added to reach a final volume of 0.25 mL. The mixture was incubated at 37°C for 1 h, and the reaction was stopped by the addition of 50 μL of H_2_SO_4_. The reaction tubes were then heated for 15 min at 60°C to decarboxylate acetolactate to acetoin. Acetoin was detected as a colored complex (A_520__nm_) formed after the addition of 0.25 mL of creatine (5 g L^-1^ freshly prepared in water) and 0.25 mL of 1-naphthol (50 g L^-1^ freshly prepared in 5 N NaOH) prior to incubation at 60°C for 15 min. The background was subtracted using control tubes in which the reaction was stopped prior to incubation.

The protein was determinated by the Bradford method (Bradford, 1976). The concentration of herbicide required to inhibit ALS activity by 50% (I_50_) was calculated from linear plots of the inhibition percentages *vs*. the logarithm of herbicide concentration as previously described (Rosario et al., 2011). The resistance factor was computed as I_50_(R)/I_50_(S). Three experiments, each performed with a separate tissue extract from a different plant, were conducted for each cultivar, and each sample at each herbicide concentration was assayed in triplicate.

### ALS Gene Sequencing

Seeds from R and S populations of *R. rugosum* were germinated on filter paper in Petri dishes after breaking the dormancy. 15 germinated seeds of R and S populations were grown in suitable pots and moved in a greenhouse. Leaf sections (200 mg) from each resistant and susceptible population at 3–4 leaf stage were taken and each leaf tissue was temporarily stored at -20°C, until use. Then the whole plants were treated with the recommended dose of TM. The individual samples from R and S populations according to the result of applying herbicide (21 days after treatment) were used for DNA extraction from the leaf material of each plant with the Speed tools kit DNA Extraction Kit Cat Plant (Biotools B & M Labs. S.A).

Because there was not any information available regarding the ALS gene sequence of *R. rugosum*, were used the same primers than those used for *Sinapis alba* (Cruz-Hipolito et al., 2013).

One 501-bp fragment (CAD domain) was amplified by a pair of primers (ALS3B: 5′-TCARTACTWAGTGCKACCATC-3′, ALS3F: 5′-GGRGAAGCCATTCCTCC-3′) and 639-bp fragment (BE domain) was amplified by sense and antisense primers (P1: 5′-GAAGCCCTCGARCGTCAAGG-3′, P2: 5′-ATA GGTTGWTCCCARTTAG-3′).

Five conserved domains of ALS gene were amplified by PCR technique with a final volume of 20 μL containing 10 ng of DNA, 0.2 mM of each sense and antisense primer pairs (detailed above for each domain), 200 μM dNTPs, 2 μL of PCR buffer, 1.5 mM MgCl_2_, and 2.5 U of Taq DNA polymerase (Genet Bio, Inc.).

The amplification cycle was that detailed in Cruz-Hipolito et al. (2013). PCR amplification products were separated on a 1.5% agarose gel (Thermo Midicell Primo EC 330), containing TBE (89 mM Tris-borate and 2 M EDTA-Na) and a 4:1 v/v DNA-charged buffer [Bromofenol blue at 0.5%, Cianol of Xilene at 0.25 and 30% glycerol (dissolved in distilled water)]. Amplified DNA fragments were purified with the use of a Speed tools PCR Clean Up Kit (Biotools, B&M Labs, Madrid, Spain), which eliminated the primers, salts, and Taq-polymerase following PCR. Sequencing of the purified genomic DNA was done in the Genomic Unit Investigation Central Service of Extremadura University, Spain.

### Statistical Analyses

The experiments followed a completely randomized design with three replicates per treatment. The experimental unit was comprised of one pot containing four plants.

Analyses of dose-response data in the whole plant and ALS activity assays were performed using the R software (drc package) by fitting the data to a non-linear log-logistic regression model as follows:

$$Y=c+\left\{(d-c)/\left[{1+\left(x/g\right)}^{b}\right]\right\}$$

Where *C* is the lower limit, *D* is the upper limit, *b* is the slope at GR_50_ (or I_50_), *g* denotes GR_50_ (or I_50_) and *x* is an independent variable representing the herbicide rate. If the C parameter was not significant with 0, C was deleted from the equation and we used three parameter logistic functions. For the calculation of the dose-response curves, the dry weight of the plants and ALS activity were taken relative to the untreated control.

The RF was calculated by dividing the determined GR_50_ (or I_50_) value of the resistant population by that of the sensitive population to determine the level of resistance of the resistant population.

The effect of population and the time, as well as their interaction in the ^14^C-tribenuron-methyl (^14^C TM) absorption, were subjected to ANOVA. The population was treated as a fixed factor while the time was considered as a random factor. The means and standard errors (average) of ^14^C-TM absorption and translocation were calculated for all parts of the plants, and the means were analyzed by different groups. For each analysis, assumptions such as equal variance and normal distribution were evaluated. When required, the LSD test at 5% probability was used for mean separation. Statistical analyses were performed using the Statistix (Analytical Software, USA) software (version 9.0).

## Results

### Physiological Properties

#### Screening Test

The response of 30 populations was investigated applying tribenuron-methyl (TM) under greenhouse conditions to determine the effect of TM on the dry weight and their survival rate. The screening test with the field rate of TM confirmed the differences between 10 populations of *R. rugosum* and Ag-R7 as an accepted susceptible population (**Table 1**). There were significant differences in the reduction of dry weight compared to the untreated control (*P* < 0.00001), the reduction being less than 20% of the untreated control (**Table 1**). R plants suffered from a little damage but they were able to recover over time. Increased sensitivity to TM was observed in 20 populations that could not survive after the application of the herbicide, whose losses of dry weight varied from between 100 and 68% compared to the control population.

**Table 1:** Effect of tribenuron-methyl at 15 g a.i ha^-1^ on dry weight, plant number of *Rapistrum rugosum* populations, visual assessment (based on EWRC score) and UTM coordinate.

*Rapistrum* population	Survived plant (% of untreated control)	Dry weight of survived plants (% of untreated control)	EWRC	UTM coordinate	
Ag-92-1	100a^∗^	85.19a	9	40S271362	40S271362
Ag-93-1	100a	87.62a	9	40S270841	40S270841
Ag-R1	100a	84.57a	9	40S270056	40S270056
Ag-R2	40c	20.3bc	2	40S269761	40S269761
Ag-R3	100a	86.67a	9	40S268706	40S268706
Ag-R4	20d	9c	1	40S268117	40S268117
Ag-R5	100a	91.2a	9	40S276142	40S276142
Ag-R6	75ab	80.25a	8	40S275735	40S275735
Ag-R7	0e	0c	1	40S275741	40S275741
Ag-R8	100a	84.71a	9	40S276130	40S276130
Ag-R9	20d	16c	2	40S276082	40S276082
Ag-R10	20d	7c	1	40S271522	40S271522
Ag-R11	20d	11c	1	40S270169	40S270169
Ag-Rr	100a	89.47a	9	40S268898	40S268898
Al-R1	20d	14c	2	40S307739	40S307739
Al-R2	20d	12c	2	40S305581	40S305581
Al-R3	0e	0c	1	40S305802	40S305802
Al-R4	20d	11.2c	2	40S303500	40S303500
Al-R5	20d	5c	1	40S311264	40S311264
Al-R6	0e	0c	1	40S311033	40S311033
G-1	100a	90.24a	9	40S266203	40S266203
G-2	100a	86.84a	9	40S267484	40S267484
G-3	20d	7c	1	40S267735	40S267735
Kr-R1	20d	15c	2	40S240284	40S240284
Kr-R2	20d	12.3c	2	40S239838	40S239838
Kr-R3	20d	17c	3	40S239503	40S239503
Kr-R4	20d	8c	1	40S244009	40S244009
Kr-R5	0e	0c	1	40S244453	40S244453
Kr-R6	20d	10.84c	2	40S244301	40S244301
Kr-R7	40c	32bc	3	40S242409	40S242409

*^∗^Lowercase letters indicate significant differences between untreated and treated plants in each population according to a LSD test (α = 0.05).*

#### TM Plant Response

In these experiments, 10 resistant populations (R) according to results of screening test, and one susceptible population (S) were used for investigation of R populations response to the increased concentration of herbicide. Dry weight values of R and S were well-fitted to the non-linear log-logistic regression model (data not shown) to estimate the effective dose that gives a 50% reduction in dry biomass (**Table 2**).

**Table 2:** Estimated non-linear regression parameters (Eq. 1) for whole-plant assays of susceptible (S) and resistant (R) populations of *R. rugosum* in response to tribenuron-methyl.

Population	Lower limit	Upper limit	Slope	GR_50_	RF
*Ag-R5*	15.58 (2.77)	100.77 (2.87)	0.67 (0.10)	71.79 (16.26)	**6.59^∗∗a^**
*G-1*	9.59 (1.40)	98.51 (3.46)	0.8 (0.13)	52.91 (9.19)	**4.85^∗∗^**
*Ag-92-1*	11.95 (2.22)	93.33 (5.62)	0.85 (0.21)	49.99 (3.56)	4.59^∗∗^
*G-2*	0	100.47 (7.62)	0.79 (0.11)	45.15 (5.60)	4.14^∗∗^
*Ag-93-1*	12.02 (1.65)	102.49 (4.53)	0.70 (0.06)	44.96 (8.76)	4.12^∗∗^
*Ag-R6*	13.17 (1.15)	98.14 (3.19)	0.91 (0.10)	35.51 (9.98)	3.26^∗∗^
*Ag-Rr*	10.53 (2.04)	101.56 (2.94)	0.94 (0.19)	30.17 (4.55)	**2.77^∗∗^**
*Ag-R3*	10.84 (2.65)	100.67 (5.18)	0.96 (0.07)	28.08 (3.34)	2.58^∗∗^
*Ag-R1*	0	101.33 (2.62)	0.95 (0.13)	27.78 (3.67)	2.55^∗∗^
*Ag-R8*	11.79 (3.06)	96.48 (1.63)	1.2 (0.20)	27.25 (2.58)	2.50^∗∗^
*S*	0	97.42 (3.65)	1.40 (0.17)	10.90 (1.47)	–

*Values in parentheses are standard errors. GR_50_ = effective rate required to reduce the response of plants to 50%. RF = Resistance factor (GR_50_ R/GR_50_ S). ^a^Level of significance probability non-linear regression model. ^∗∗^P = 0.01.*

**FIGURE 1:**
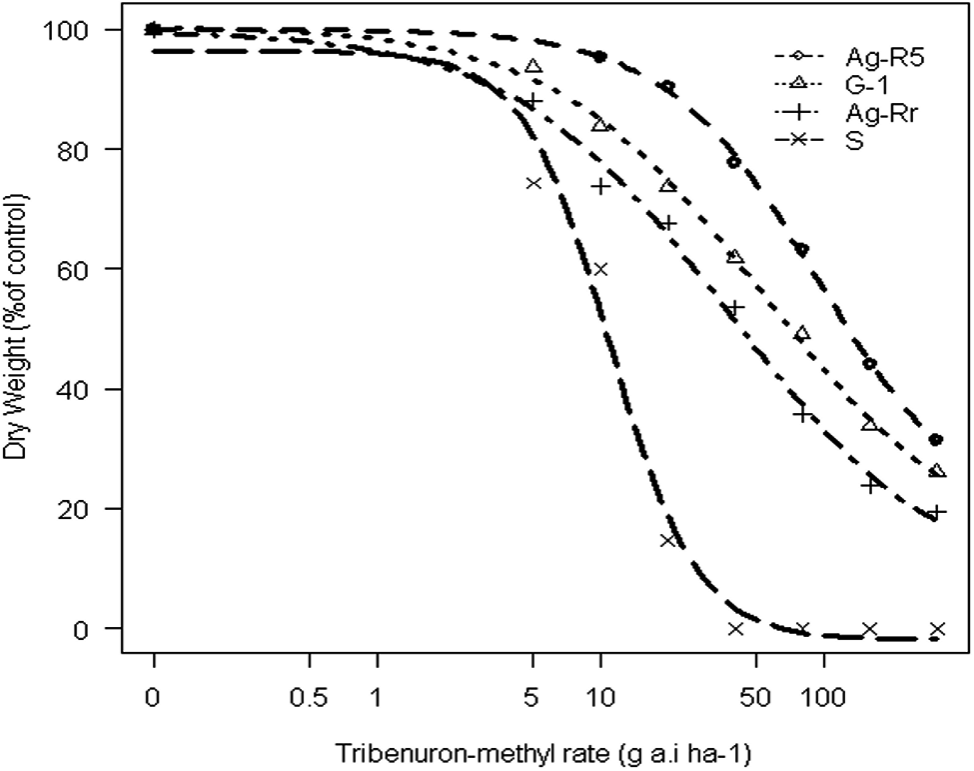
**Response of dry weight of susceptible and resistant populations of *Rapistrum rugosum* to different concentrations of the tribenuron-methyl**.

The concentration of TM that led to 50% inhibition shoot growth in control treatment was 10.90 g a.i. ha^-1^, whereas for R populations it caused 2.5–6.6 fold more resistance relative to the S one. In short, the whole-plant bioassay experiment confirmed that the selected biotypes from the screen test were resistant to TM (**Table 1**). Due to the large number of resistant populations, three of them were selected (Ag-R5, G-1 and Ag-Rr) and one susceptible (Ag-R7, from now named as S) to resistance mechanisms research (**Table 2**). **Figure 1** shows dose-response curves of these populations. Other experiments were conducted on the three populations as being representative of all resistant populations.

#### TM Spray Retention

In our cases, the studied populations showed no significant differences for retention of labeled TM, according to ANOVA (*P* = 0.2161). The results showed the rate of retention of herbicide as 0.89, 0.92, 0.96, and 1.09 ml g^-1^ dry matter for Ag-R5, G-1, Ag-Rr, and S populations, respectively (data not shown), in which the rates were not statistically significant. Therefore, we have concluded that this physical parameter is not responsible for the observed resistance in *R. rugosum* populations.

### Non-target-site Resistance

#### ^14^C-TM Absorption and Translocation

**FIGURE 2:**
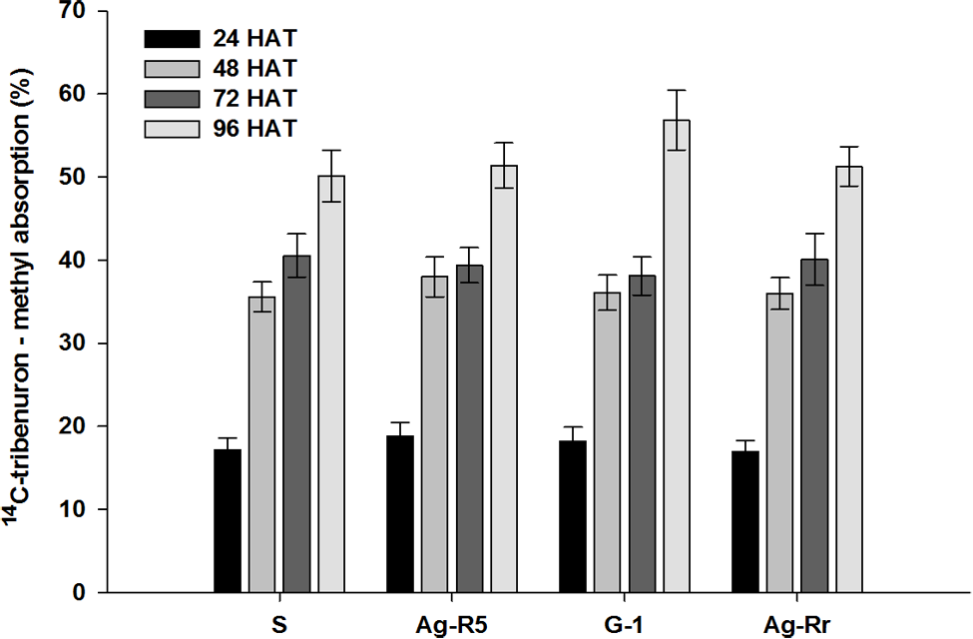
**Absorption of ^14^C-tribenuron-methyl in the susceptible and resistant populations of *R. rugosum***.

**FIGURE 3:**
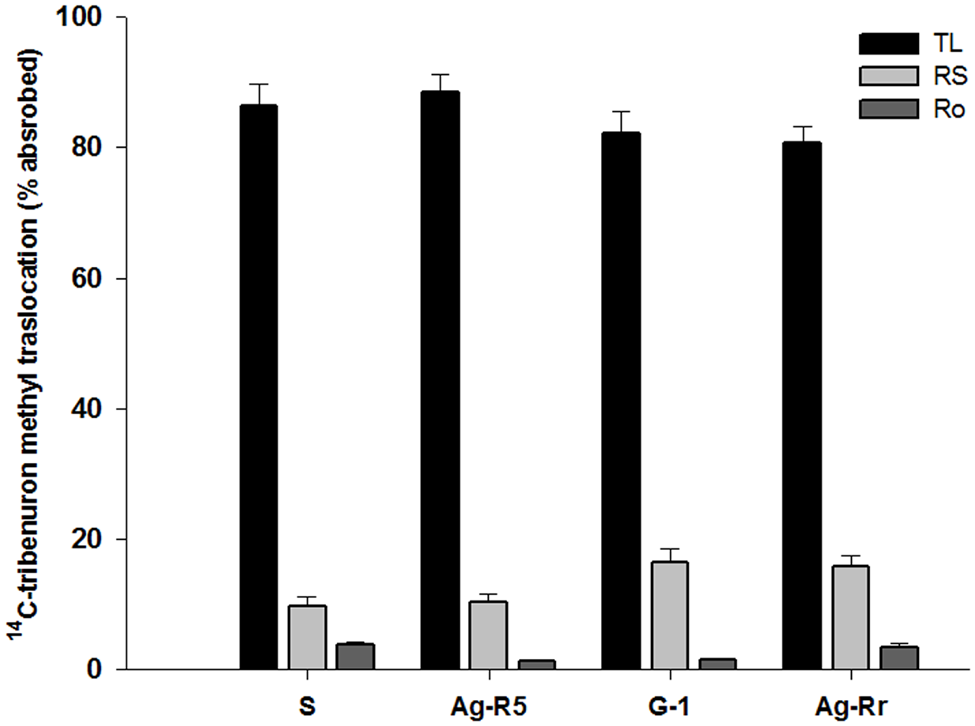
**Percent of translocated ^14^C-tribenuron-methyl into three different parts of four *R. rugosum* populations**.

Absorption was significant in each population at the different times (*P* < 0.0001). There were no significant differences (*P* > 0.05) between the R and S populations in the herbicide absorption into the leaf tissue at the same times (**Figure 2**). Absorption of ^14^C-TM in both sensitive and resistant populations after treatment increased. At 48 (HAT), the absorption was over 50% of the maximum amount of the applied herbicide. The ^14^C-herbicide moved from treated leaf (TL), to the remainder shoots (RS) and roots (Ro). From **Figure 3** it can be observed that 3.43, 1.42, 1.22, and 3.82% of labeled herbicide moved to Ro, and 15.74, 16.43, 10.23, and 9.78% translocated to RS from the TL in Ag-Rr, G-1, Ag-R5 and susceptible populations, respectively. However, a larger amount of ^14^C-tribenuron-methyl applied was retained on the TL (80.83, 82.15, 88.55, and 86.40% for the R and S mentioned populations, respectively; **Figure 3**).

**FIGURE 4:**
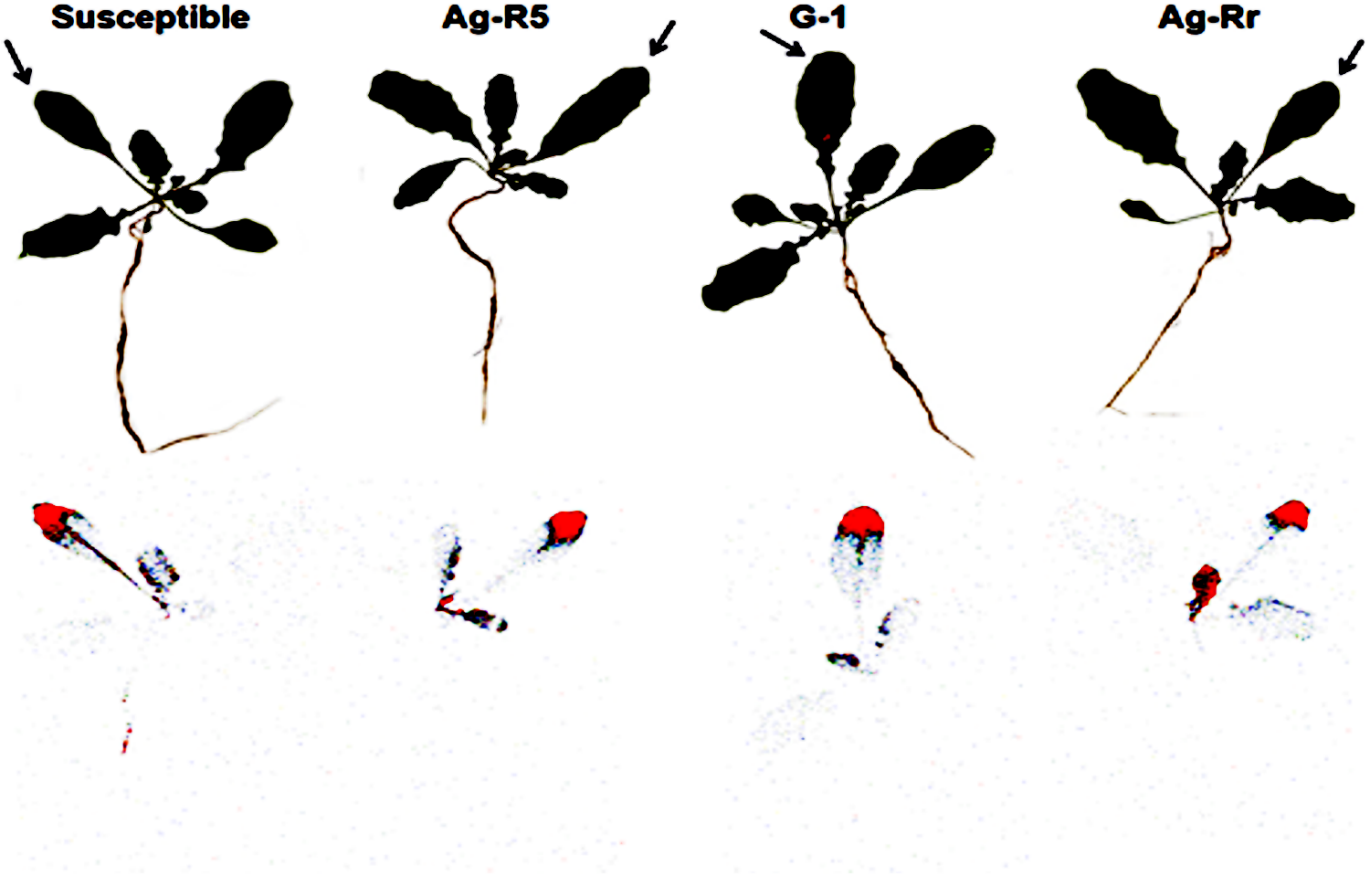
**Digital images (upper plants) and autoradiographic images (lower plants) showing ^14^C-tribenuron-methyl translocation throughout plant tissues of R and S populations of *R. rugosum*, 72 HAT.** The arrows indicate the site of ^14^C-tribenuron-methyl application.

The autoradiography images obtained by the Cyclone at 72 HAT showed that the ^14^C-TM translocation was at the same level in resistant and susceptible populations. In addition, a small amount of this herbicide was transferred to RS and Ro from TL (**Figure 4**). The results demonstrated that the absorption and translocation of the herbicide did not play any effective role in the resistance to ALS inhibitor herbicides developed by these populations.

#### ^14^C-TM Metabolism

Qualitative and quantitative differences were found between the populations. The amount of metabolites between each population was very different (**Table 3**). The three metabolites (MM, OH-MM, and conjugate-MM) were detected at 96 HAT only in two populations (Ag-R5 and G-1), while in the population Ag-Rr only two metabolites were found and in the S population only one (**Table 3**). With respect to the amount of the different metabolites between the populations, the amount of the ^14^C-TM found in Ag-Rr and S plants was higher than in Ag-R5 and G-1 plants (**Table 3**). The *N*-demethylation of TM to form MM (another active SU herbicide) was similar in all plants and there were no significant differences.

**Table 3:** Relative percentage of tribenuron-methyl and its metabolites of different resistant (R) and susceptible (S) *R. rugosum* populations at 96 HAT.

Population	TM^a,b^	MM^a,c^	OH-MM^a^	Conjugated-MM^a^
Ag-R5	31.9^a^ (1.2)	10.3^a^ (2.3)	22.4^a^ (1.2)	35.4^a^ (6.7)
G-1	53.6^b^ (6.8)	15.4^a^ (5.4)	18.4^b^ (0.8)	12.6^b^ (1.8)
Ag-Rr	81.7^c^ (9.1)	15.2^a^ (6.1)	3.1^c^ (0.1)	nd
S	88.4^c^ (6.3)	11.6^a^ (3.6)	nd	nd

*^a^Means within a column followed by the same letter are not significantly different at the 5% level as determined by the LSD test. Values in parentheses are standard errors. TM^b^, tribenuron-methyl; MM^c^, metsulfuron-methyl; n.s, non-significant.*

The MM metabolite was rapidly degraded, first through the hydroxylation of the phenyl ring generating the OH-MM, which was not present in S plant but was in the other three resistant populations in significant amounts, i.e., 22.4, 18.4, and 3.1% for Ag-R5, G-1, and Ag-Rr, respectively. The third metabolite, conjugate-MM, was formed by conjugation of OH-MM with carbohydrates. Conjugate-MM levels did not appear in Ag-Rr and S plants and its level was decisive and higher in Ag-R5 than in G-1 (**Table 3**). Both OH-MM and conjugate-MM are non-toxic for plants. Due to the differences found in the amounts of metabolite between the populations, it is likely that resistance of Ag-R5 and G-1 plants was based up increased TM metabolism.

### Target-Site Resistance

#### ALS Activity Assay

The data for each population showed a significant fit (*P* < 0.01) to the logistic equation (**Table 4**). The RF [(I_50_ R/I_50_ S)] for Ag-R5, G-1 and Ag-Rr populations with TM were 30.53, 38.19, and 28.60 fold higher than the S population, respectively. Following this order, the RFs with florasulam were 17.29, 12.38 and 11.52, with bispyribac-sodium they were 6.81, 4.96 and 5.83, with flucarbazone they were 9.87, 5.02 and 5.47, and finally with imazamox they were 0.90, 0.82, and 0.64 fold higher than the S population (**Table 4**).

**Table 4:** Effective concentration required to reduce the ALS activity to 50% and resistance factor (RF: I_50_ R/I_50_ S) in *R. rugosum* using different herbicides concentration.

Herbicide	Population	I_50_ (μM)	RF
Tribenuron-methyl	Ag-R5	2152. 93 (100.1)	30.53^∗∗a^
	G-1	2692.8 (314.9)	38.19^∗∗^
	Ag-Rr	2016.41 (173.2)	28.60^∗∗^
	S	70.50 (19.4)	–
Florasulam	Ag-R5	1152.90 (94.9)	17.29^∗∗^
	G-1	825.64 (101.6)	12.38^∗∗^
	Ag-Rr	767.87 (76.6)	11.52^∗∗^
	S	66.66 (11.7)	–
Bispyribac-sodium	Ag-R5	399.19 (56.8)	6.81^∗∗^
	G-1	290.65 (49.9)	4.96^∗∗^
	Ag-Rr	342.03 (50.5)	5.83^∗∗^
	S	58.59 (11.8)	–
Flucarbazone	Ag-R5	540.37 (73.0)	9.87^∗∗^
	G-1	275.12 (46.7)	5.02^∗∗^
	Ag-Rr	299.74 (47.7)	5.47^∗∗^
	S	54.77 (10.8)	–
Imazamox	Ag-R5	220.97 (48.6)	0.90^n.s^
	G-1	201.01 (41.9)	0.82^n.s^
	Ag-Rr	157.17 (30.3)	0.64^n.s^
	S	244.17 (41.2)	–

*Values in parentheses are standard errors. ^a^Level of significance probability non-linear regression model. n.s, non-significant. ^∗∗^P = 0.01.*

The specific *in vitro* activity of the ALS enzyme obtained from shoot R (Ag-Rr, G-1, Ag-R5) and S of *R. rugosum* tissue was similar, with no significant differences (418, 398, 405, and 441 nmol acetoin per mg protein per hour, respectively). ALS activity data of all populations were analyzed with the same statistical procedure used in the dose response experiments.

**FIGURE 5:**
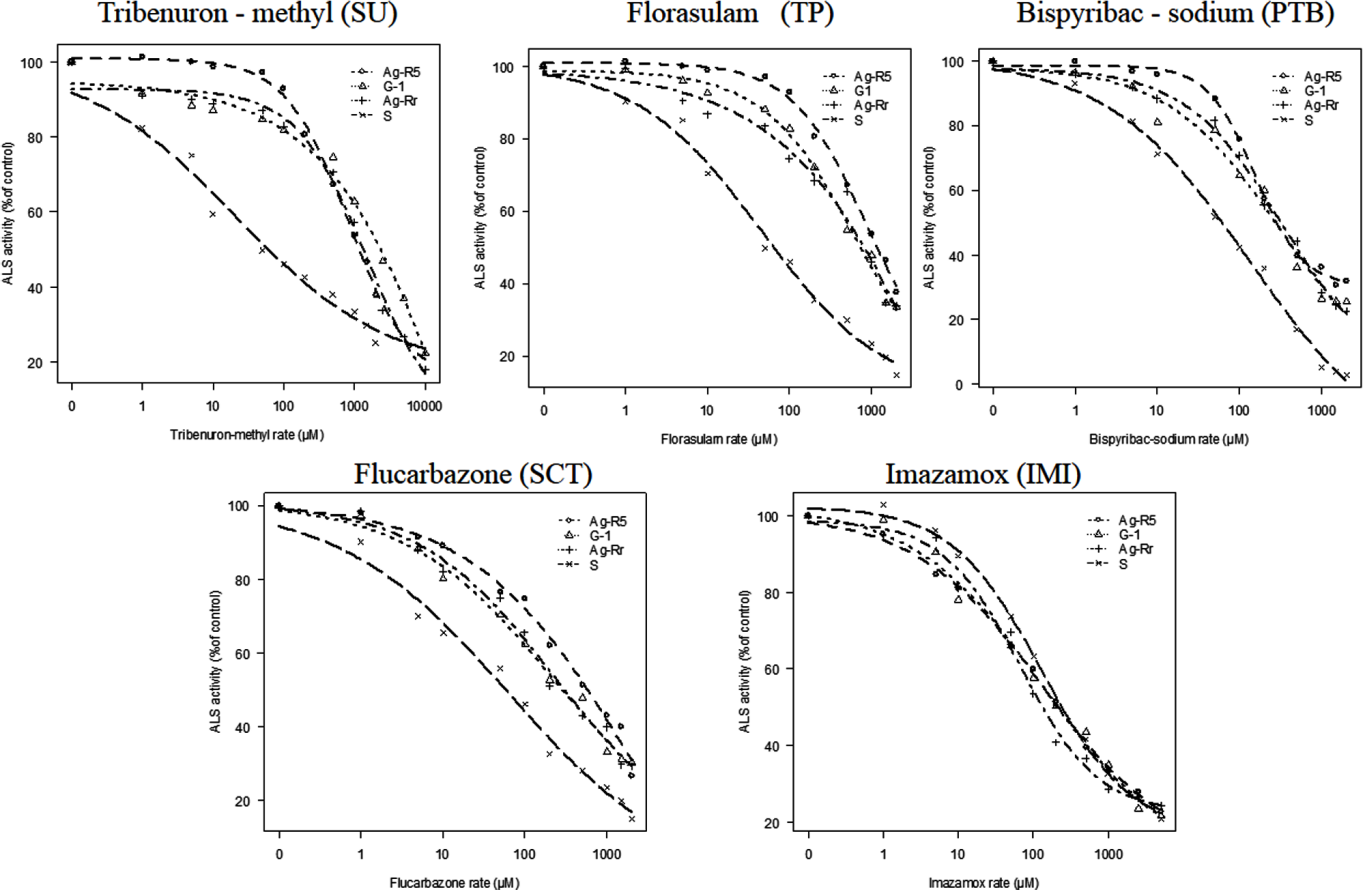
**Effects of five ALS inhibitor families on ALS activity of resistant (R) and susceptible (S) *R. rugosum* populations expressed as a percentage of the untreated control.** Actual data are mean of six replications.

Also, we saw that the R plants were resistant to four evaluated ALS-inhibiting chemical families (**Figure 5**). However, *R. rugosum* showed no cross-resistance to the imidazolinone family (**Table 4**).

#### ALS Gene Sequencing

**FIGURE 6:**
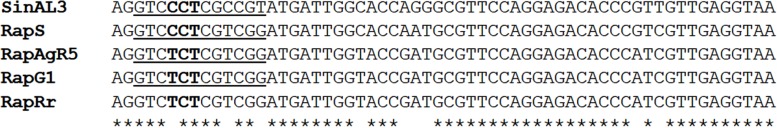
**Sequence alignment of the CAD domain of the ALS gene in the four populations of *R. rugosum* (AGR5, G1, AGRr, and S) compared with a susceptible population of *Sinapis alba* (SINAL3).** Proline by serine amino-acid substitution occurred in position 197. There are no amino acid changes in the C, D, B, and E domains of the *R. rugosum* ALS gene compared to S plants. Numbering of the ALS gene sequence alignment of *Arabidopsis thaliana*.

Biochemical and molecular data showed a substitution Pro197 to Ser197 in R populations (**Figure 6**). This mutation resulted in having a high level of resistance to tribenuron-methyl (SU), a relatively low level of resistance to florasulam (TP), flucarbazone (SCT), and bispyribac-sodium (PTB) herbicide but no resistance to imazamox (IMI) herbicide.

## Discussion

### Physiological

In Iran, wheat is one of the principal agricultural productions which is mainly grown in monoculture and the only used methods to remove the weeds is the herbicides application. In our study, the responses of the *R. rugosum* populations collected after treatment with the field rate of TM confirmed that some of these populations were resistant to several herbicides of the ALS-inhibitors group. The first case of a chlorsulfuron-resistant *R. rugosum* population was detected in Australia but its study is incomplete (Adkins et al., 1997). The resistance level obtained for the different populations of *R. rapistrum* was lower than that obtained for *S. alba* found in a wheat field in Spain, or in the case of *Myosoton aquaticum* selected by TM treatment for 5 years in China (Rosario et al., 2011; Liu et al., 2015).

The effectiveness of an herbicide can be determined by the maximum amount of the herbicide that can be captured by the target weeds. In some cases, differences in spray retention have been found between species (Chachalis and Reddy, 2005; González-Torralva et al., 2010) and biotypes of the same species resistant to herbicides (Michitte et al., 2007; Rosario et al., 2011; Cruz-Hipolito et al., 2013). However, in our TM spray retention assay we did not find any significant differences between the different R and S populations of *R. rugosum*.

These results suggest that resistance is not due to differences in the composition of the cuticular wax, that lead to reducing the contact angle of sprayed droplets and thus lowering the herbicide retention potential and ability on the leaves and/or differences in the leaf angle, which in turn leads to the difficulties in the herbicide droplets reaching the leaves (De Prado et al., 2005; Alcántara et al., 2016).

### Non-target-site Resistance

The decreased level of herbicide absorption has been suggested, as being a potential mechanism of resistance in a weed biotype, but the increased level of herbicide absorption observed in resistant populations does not appear to bring any particular advantage to the survival of these populations following the herbicide treatment (Devine and Shimabukuro, 1994; Devine, 2002).

There are no demonstrations of the differential herbicide translocation playing the main role in resistance to ALS inhibitor herbicides (Cruz-Hipolito et al., 2013; Riar et al., 2013; Yu and Powles, 2014b). In some cases, they could be active especially where differential translocation seems to be involved, but, here there was no evidence for the nature of herbicide molecules being translocated (either in the parent herbicides or in their metabolites), so that those cases do not correspond to our analysis.

The results demonstrated that the absorption and translocation of the herbicide do not play any effective role in the resistance to ALS inhibitor herbicides developed by these populations, which is in line with the earlier results of other researchers (Osuna et al., 2003; Rosario et al., 2011; Owen and Powles, 2014).

Knowledge of the mechanism of resistance is very important for managing a successful field control. Also, employing herbicides with different modes of action would be required. Metabolic resistance often confers resistance to herbicides of different chemical groups and sites of action and can extend to new herbicide(s). This is a very dangerous fact because our options for weed control management thus diminish.

Farmers have been using low doses of herbicides, which leads to selecting populations that are able to metabolize the herbicide. So, after a while, the farmers are faced with control failures and increase the herbicide dose, which results in the selection of resistant mutations in the given gene, so that the plants may have the target base and non- target base of resistance mechanism (Yu and Powles, 2014a,b).

The resistance phenomena will bring about ecological consequences such as changes in the plant flora, the possibility of resistance gene flow to the close relatives or other possible environmental aftermaths due to the increased use of herbicides for weeds resistant control purpose. Moreover, the use of several types of herbicides on the weeds can results in multiple resistance of the plants. Non target- site resistance to ALS has been rarely documented in dicot weeds, the last case has been described in waterhemp (*Amaranthus tuberculatus*) by Guo et al. (2015).

There are some cases in which metabolism has been described as being the dominant mechanism of ALS resistance (Saari et al., 1990, 1994; Osuna et al., 2003; Park et al., 2004). Resistant rigid ryegrass biotypes metabolized chlorsulfuron more rapidly than the susceptible biotype (Christopher et al., 1992; Yu and Powles, 2014b). Also, a resistant biotype of wild mustard metabolized ethametsulfuron-methyl more rapidly than the S biotype (Veldhuis et al., 2000). Owen et al. (2012) showed that six *Bromus rigidum* populations have a low-level resistance to the ALS-inhibiting SU herbicides, but are able to be controlled by other herbicide modes of action. A low-level, malathion-reversible resistance, together with a sensitive ALS, strongly suggested that a non-target-site enhanced metabolism was the mechanism of resistance. Jeffers et al. (1996) observed that a resistant (R) biotype of wild mustard was 48-fold more resistant to ethametsulfuronmethyl than a susceptible (S) wild mustard. Furthermore, on the basis of the lack of cross-reactivity of this biotype to other SU herbicides, they suggested that resistance of this biotype to ethametsulfuronmethyl might be due to enhanced metabolism. Researchers have shown resistance to SU herbicides in the grass weeds *Lolium rigidium* and *Alopecurus myosuroides* is due to increased metabolism (Preston et al., 1996; Menendez et al., 1997). Hall et al. (1992) and Lichtner et al. (1995) reported rapid metabolism of ethametsulfuron-methyl in tolerant commercial brown mustard and oilseed rape as opposed to S wild mustard.

### Target-Site Resistance

In this work, *in vitro* assays R populations (Ag-R5, G-1, and Ag-Rr) shown resistance to four ALS family herbicides (SU, PTB, SCT, and TP) but these populations were susceptible to IMI. This cross-resistance to ALS inhibitor herbicides has been due a one punctual mutation at Pro197Ser, as has been detected by other dicot weeds (Cruz-Hipolito et al., 2013; Yu and Powles, 2014a; Liu et al., 2015).

Amino acid substitutions of Pro197 by Ser, His, Leu, Ala, or Thr have been observed to result in resistance to SU herbicide

(Cui et al., 2011; Satoshi et al., 2014; Deng et al., 2015). Multiple mutations including Pro to Arg, Leu, Gln, Ser, or Ala have been identified in *Kochia*-resistant biotypes to confer a high level of resistance to SU herbicides (Saari et al., 1994; Guttieri et al., 1995).

A Pro to Ser change was identified in resistant the *Sinapis arvensis* (Warwick et al., 2005; Cruz-Hipolito et al., 2013). The replacement of Pro with Ala, Ser, or Gln is involved in resistance to SU herbicides in seven of 14 resistant biotypes of *Lindernia* sp. (Uchino and Watanabe, 2002). Pro substitution by four different amino acids Ala, His, Ser, and Thr was reported in eight resistant wild radish populations (Yu et al., 2003). The relationship between the amino acid substitution and cross-resistance pattern has been established for a few biotypes. A Pro197 to Ser or Ala mutation was demonstrated to result in resistance to SU, PTB, and TP but not IMI herbicides in *Conyza canadensis, S. alba*, and *Myosoton aquaticum* (Zheng et al., 2011; Cruz-Hipolito et al., 2013; Liu et al., 2015). Although amino acid substitutions occurring at specific sites of the ALS gene have been documented for particular cross-resistance patterns (Saari et al., 1994; Warwick et al., 2005; Zheng et al., 2011), the different amino acid substitutions at the same mutation site may confer different levels of resistance to one of specific herbicide or a group of them.

The SU herbicides make multiple hydrophobic interactions with ALS as well as hydrogen bonds. The substitutions of acidic, basic, and uncharged amino acids for Pro197 all provide resistance to SU herbicides (McCourt et al., 2005). These substitutions must affect the size or shape of the binding pocket for the herbicides rather than just influencing hydrophobic interactions with the herbicides (Stewart, 2009).

The high frequency of proline-site mutation is because the changes at this proline site are not linked with major fitness penalties (Stewart, 2009). Also, the extensive worldwide use of SU herbicides is likely the reason for the regular appearance of amino acid substitutions at Pro197 within the resistant ALS gene.

## Conclusion

Different TM-resistance levels between the *R. rugosum* populations could be caused by enhanced Cyt. P_450_ metabolism and/or punctual ALS mutation. Furthermore, this mutation might be responsible to cross-resistance to four ALS family groups. The multiple resistance mechanisms result in high complexity and leading to the inheritance which are more difficult to control and determine (Yu and Powles, 2014a,b).

In this research, Point mutation of ALS (Target site) contributes to low level of TM resistance in *R. rugosum*. Addition of enhanced metabolism (Non-target site), increased the level of resistance depending on enhanced rate of TM metabolism. As seen at **Tables 2** and **3**, Ag-Rr population without non-target site resistance, displayed lower RF in the greenhouse experiments than two other populations (Ag-R5 and G-1). Ag-R5 has higher GR_50_ compare with two other populations due to increased non-toxic metabolites in the other hand, more metabolizing the TM.

Because metabolic resistance is unpredictable and can trigger herbicide resistance with similar mechanisms or even different MOA, including herbicides never used, delaying and /or stopping the spread of the fast evolution of *R. rugosum* could be the priority. Quarantining weed infested areas immediately might be the strategy required, as well as encouraging the training of growers and crop consultants in tackling herbicide resistant weeds and adopting herbicide and crop rotations and other agronomic and integrated management practices to reduce herbicide selection pressure, preventing or delaying the development of *R. rugosum* resistance. Nowadays, for prevention of the further spread of resistance, crop rotations and utilization of alternative herbicides are greatly recommended. Alternative herbicides in Iran market that can be used for control of broad leaf weeds in wheat fields include auxin-type herbicides (such as, 2-4-D,2-4-D+MCPA, dichlorprop-*p* + MCPA + mecoprop-p, 2-4-D + dicamba), PS II inhibitors (such as, bromoxynil, isoproturon + diflufenican or panter) and herbicides combined (bromoxynil + MCPA). Based on the climate and characters of the region, canola also can be used for crop rotation purpose with wheat or the selective herbicides which are not common with wheat can be employed. There are huge initiatives to control plant resistance in different regions of Iran.

## Author Contributions

ZH, JG, and HS carried out the in-field seed screenings; ZM, RA, PF, AR-D, and RDP performed the TM plant response, TM spray retention, and ^14^C-TM absorption and translocation; ZH, AR-D, and RDP performed the ^14^C-TM Metabolism, and ALS enzyme activity; ZH, JG, MO, and RDP performed the ALS gene sequencing.

## Conflict of Interest Statement

The authors declare that the research was conducted in the absence of any commercial or financial relationships that could be construed as a potential conflict of interest.
